# Inhibition of Wnt/β-Catenin Signaling by a Soluble Collagen-Derived Frizzled Domain Interacting with Wnt3a and the Receptors Frizzled 1 and 8

**DOI:** 10.1371/journal.pone.0030601

**Published:** 2012-01-27

**Authors:** Ismaïl Hendaoui, Elise Lavergne, Heun-Sik Lee, Seong Hyun Hong, Hak-Zoo Kim, Christelle Parent, Nathalie Heuzé-Vourc'h, Bruno Clément, Orlando Musso

**Affiliations:** 1 Institut National de la Santé et de la Recherche Médicale, Unit 991, Liver Metabolisms and Cancer, Rennes, France; 2 Université de Rennes 1, Rennes, France; 3 INSERM, Unit 618, Proteases and Pulmonary Vectorization, Tours, France; 4 Gyeonggi Institute of Science and Technology Promotion, Gyeonggi Bio-Center, Suwon-city, South Korea; National Cancer Center, Japan

## Abstract

The Wnt/β-catenin pathway controls cell proliferation, death and differentiation. Several families of extracellular proteins can antagonize Wnt/β-catenin signaling, including the decoy receptors known as secreted frizzled related proteins (SFRPs), which have a cysteine-rich domain (CRD) structurally similar to the extracellular Wnt-binding domain of the frizzled receptors. SFRPs inhibit Wnt signaling by sequestering Wnts through the CRD or by forming inactive complexes with the frizzled receptors. Other endogenous molecules carrying frizzled CRDs inhibit Wnt signaling, such as V3Nter, which is proteolytically derived from the cell surface component collagen XVIII and contains a biologically active frizzled domain (FZC18) inhibiting *in vivo* cell proliferation and tumor growth in mice. We recently showed that FZC18 expressing cells deliver short-range signals to neighboring cells, decreasing their proliferation *in vitro* and *in vivo* through the Wnt/β-catenin signaling pathway. Here, using low concentrations of soluble FZC18 and Wnt3a, we show that they physically interact in a cell-free system. In addition, soluble FZC18 binds the frizzled 1 and 8 receptors' CRDs, reducing cell sensitivity to Wnt3a. Conversely, inhibition of Wnt/β-catenin signaling was partially rescued by the expression of full-length frizzled 1 and 8 receptors, but enhanced by the expression of a chimeric cell-membrane-tethered frizzled 8 CRD. Moreover, soluble, partially purified recombinant FZC18_CRD inhibited Wnt3a-induced β-catenin activation. Taken together, the data indicate that collagen XVIII-derived frizzled CRD shifts Wnt sensitivity of normal cells to a lower pitch and controls their growth.

## Introduction

The Wnt/β-catenin pathway controls cell fate through regulation of cell proliferation and death, migration, differentiation and metabolism [1]. Pathway activation involves interaction of Wnt ligands with cell surface Frizzled receptors and LRP5/6 co-receptors. This disrupts the *Adenomatous polyposis coli* (APC)-axin complex, thus halting proteasomal degradation of β-catenin, which is stabilized and interacts with T-cell factor (TCF) transcription factors, displacing repressors and recruiting activators of target gene expression.

The bioavailability of Wnts at the cell surface is regulated by several families of extracellular proteins. Heparan sulfate glycosaminoglycans control Wnt diffusion, thus enhancing interaction of Wnt ligands with Frizzled receptors [2]. Antagonists include members of the *Dickkopf* (DKK) family that block canonical signaling by binding to LRP5/6, thereby disrupting the Wnt-induced Frizzled-LRP5/6 complex [3]. Wnt inhibitory factor-1 (WIF-1) binds directly to Wnts, altering their ability to interact with the receptors. The extracellular decoy receptors known as *secreted frizzled-related proteins* (SFRPs) have a frizzled *cysteine-rich domain* (CRD) structurally similar to the extracellular Wnt-binding domain of the frizzled receptors. Frizzled CRDs contain 10 cysteines at conserved positions, which form a highly conserved 3D structure, bind Wnts and form homodimers or heterodimers [4]. Thus, SFRPs can modulate Wnt signaling by sequestering Wnts through the CRD or by acting as dominant-negative inhibitors, forming inactive complexes with the frizzled receptors [5]. In addition, engineered SFRP-like proteins such as the soluble CRD of the receptor Frizzled 8 bind Wnt3a and inhibit autocrine Wnt signaling and tumor growth in mice carrying teratomas [6].

**Figure 1:**
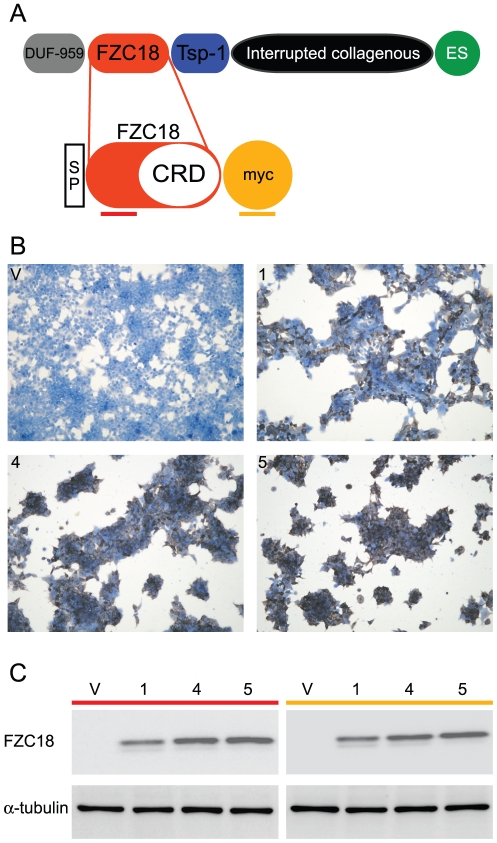
Stable expression of FZC18 in HEK293T cells. (A) Schematic structure showing the variant 3 of collagen XVIII containing DUF-959, FZC18, Tsp-1 (thrombospondin-1) and ES (endostatin) domains and the FZC18 expression vector. *Interrupted collagenous* indicates multiple triple helices (collagenous sequences) interrupted by globular domains. Thick horizontal lines indicate the antibodies used. *SP*, signal peptide; *CRD*, Cysteine-Rich Domain; *myc*, myc epitope tag. (B) HEK293T cells stably expressing FZC18 (batches 1; 4; 5) or empty vector (V) were fixed, permeabilized and immunostained with anti-myc, followed by peroxidase-conjugated antibodies (brown). Cells were counterstained with hematoxylin (blue). Original magnification: ×100. Images were acquired on an Olympus BX60 microscope. (C) Immunoblot with anti-FZC18 *(red)* and anti-myc *(yellow)* antibodies in HEK293T cell batches (1; 4; 5) stably expressing FZC18 or empty vector (V). α-tubulin is a loading standard.

In addition to SFRPs, other endogenous molecules carrying frizzled CRDs inhibit Wnt signaling. Among them, V3Nter is a cell surface polypeptide that inhibits tumor growth and switches off the β-catenin target gene expression signature *in vivo* [7], [8]. V3Nter is proteolytically derived from the cell surface extracellular matrix component collagen XVIII [7], [9], [10] and contains a biologically active frizzled domain (FZC18) (Figure 1A) [7]. The CRD in the FZC18 domain is highly conserved in frog, mouse and man, all 10 cysteines and the number and type of intervening amino-acids being fully conserved [9]. Indeed, we previously showed a 100% probability that the predicted 3D model of FZC18_CRD matches the 3D structure of mouse SFRP3 and FZD8 CRDs [7]. In human liver cancer, endogenous collagen XVIII is proteolyzed, releasing the FZC18 precursor V3Nter. We have shown that low FZC18 protein expression in liver cancer correlated with markers of high Wnt/β-catenin activity and *vice versa* [7].

In this work, we show that low concentration soluble FZC18 interacts with Wnt3a and with the receptors FZD1 and FZD8 in a cell-free system. Consequently, FZC18 reduces cell sensitivity to Wnt3a and inhibits Wnt/β-catenin signaling. In line with these findings, FZC18 inhibitory effects were partially rescued by the expression of FZD1 and FZD8 receptors, but enhanced by expression of FZD8_CRD-GPI, a cell-membrane-tethered chimeric FZD8_CRD. Finally, we produced high-yield soluble recombinant human FZC18_CRD-Fc fusion protein, which inhibited Wnt3a-induced β-catenin activation *in vitro*.

## Materials and Methods

### cDNA Clones

Human Igκ-FZC18-myc/pSecTag2 (carrying an Igκ signal peptide) and mouse Wnt3a-V5/pCDNA3.1 mammalian expression vectors, Super8•Topflash and Super8•Fopflash CRT reporters, Cyclin D1 promoter reporter D1Δ-944pXP2 and the normalization Renilla luciferase vector pGL4.70[*hRluc*] were previously described [7]. The episomal expression vector pCEP-PU was from T. Sasaki [11]. Igκ-FZC18-myc was transferred from pSecTag2 to pCEP-PU by PCR synthesis of an 875-bp fragment carrying 5′-NheI and 3′-BamHI restriction sites in the forward and reverse primers, respectively. The PCR fragment was ligated into pCRII-Topo-TA (Invitrogen), excised and subcloned into pCEP-PU. Mouse FZD8_CRD-myc/pcDNA3, mFZD8_CRD-Fc/pRK5 [12] (Addgene plasmid 16689) and empty pRK5 plasmid were from X. He, mFZD8_CRD-myc-GPI was from J. Nathans [13], mFZD8-myc receptor/pEF1A [14] was from R. Nusse, pEF1/myc-his was from Invitrogen and rat FZD1-myc (Addgene plasmid 16798) was from R. Moon. Human FZC18_CRD was PCR cloned into BamHI and KpnI in pIDZ-Fc in frame with an Igκ signal sequence and a C-terminal human IgG Fc tag for affinity purification. A thrombin cleavage site was included to allow removal of the Fc tag. The sequences of primers were: forward, 5′-GGG GGA TCC GCC CTG CTC GGG GCT GAC-3′; reverse #1, 5′-GGG CTC GAG AGA TCC ACG CGG TAC CAG TGC AGC CGG CCC AAT GAG-3′; reverse #2, 5′-GGG CTC GAG TGC AGC CGG CCC AAT GAG-3′. Constructs were stably transfected in DHFR-deficient CHO cells with Effectene transfection reagent (Qiagen), and clones selected in media containing G418 (500 µg/ml, Sigma) and lacking hypoxanthine and thymidine. All cDNAs were checked by automatic sequencing (Sequencing Facility, Rennes University Hospital, France).

### Biological Activity of Soluble FZC18

Collection of conditioned media (CM) from parental L cells (control CM) and Wnt3a CM was performed as recommended by ATCC and by R. Nusse lab website. For other CM, HEK293 EBNA cells were seeded at 2.2×10^6^ cells/10 mm dishes and transfected with either FZC18-myc/pCEP-PU, mFZD8_CRD-myc/pcDNA3, mFZD8_CRD-Fc/pRK5 or with the respective empty vectors and, 24 hr later, fresh media were replaced by DMEM (4.5 g/l glucose) without phenol red or FCS (Invitrogen). Conditioned media were collected 48 hr later, centrifuged at 450 *g* and filtered (0.2 µm). To obtain recombinant FZC18_CRD, conditioned media from hFZC18_CRD-Fc clones were screened for protein expression by ELISA and positive clones were confirmed by Western blot analysis using anti-human IgG-Fc antibody. The positive clones were further adapted to CD OptiCHO medium supplemented with 8 mM L-Glutamine. hFZC18_CRD-Fc producing cells were seeded into spinner flasks at 2×10^5^ cells/ml and incubated at 37°C and 5% CO_2_ with agitation at 80 rpm in humidified air for 10 days. The medium was collected, cleared by centrifugation, filtered (0.45 µm) and stored at 4°C until purification. The samples were loaded on a protein A column following equilibration with 20 mM sodium phosphate, 20 mM sodium citrate, pH 7.5. The column was washed with the same buffer until effluent absorbance returned to baseline. The bound proteins were eluted with 20 mM sodium phosphate, 100 mM sodium citrate, pH 2.5 followed by rapid neutralization by adding 0.1 volume of 1 M Tris-hydrochloride, pH 9.0. The yield of the purified proteins was approximately 1∼5 mg/l and purity was over 40%, as estimated by sandwich ELISA using anti-Fc antibody (Abcam AB1927) for capture and peroxidase-conjugated secondary antibody (Sigma A0170) for detection. Purified proteins were stored at −80°C until use.

### Immunological Methods

Coimmunoprecipitations were done by incubating either FZC18-myc or FZD8_CRD-myc pre-cleared CM with recombinant mWnt3a (100 ng/ml; 2.7 nM) and either mouse anti-myc or mouse IgG_1_ (Dako) on a rotary wheel at 4°C overnight. Then, protein G magnetic beads (New England Biolabs), saturated overnight in protein extracts from 293EBNA cells in RIPA buffer (TrisHCl 50 mM, pH 7.4; 1% Triton-X-100; 25 mM Hepes; 150 mM NaCl; 0.2% Sodium deoxycholate, 5 mM MgCl_2_), were added to immunocomplexes and incubated at 4°C, for 3 hr. After washing in RIPA buffer, complexes were eluted in denaturing sample buffer, resolved by 10% PAGE-SDS and immunoblotted. For reverse coimmunoprecipitation experiments, either rabbit anti-mWnt3a (C64F2, Cell Signaling) or rabbit IgG (Dako) was incubated either with recombinant mWnt3a plus FZC18-myc CM or with recombinant mWnt3a plus FZD8_CRD-myc CM. Coimmunoprecipitation of FZC18-myc with either recombinant mFZD1_CRD-Fc (100 ng/ml) or mFZD8_CRD-Fc CM was done as described above, using protein G magnetic beads binding the Fc tags. Immunoblots were performed with mouse anti-myc (Invitrogen) and with monoclonal rat anti-FZD1_CRD or anti-FZD8_CRD antibodies (R&D). Signal from immunoblots was detected by enhanced chemiluminiscence, as described [7].

## Results

### The frizzled domain of collagen XVIII inhibits cell proliferation and DNA synthesis

**Figure 2:**
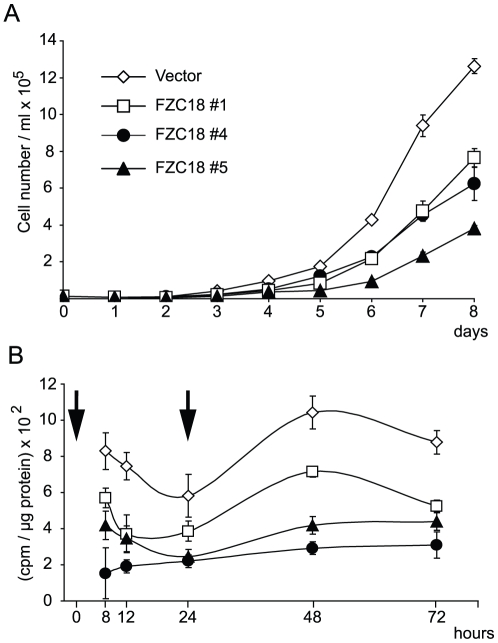
FZC18 inhibits cell proliferation and DNA synthesis. (A) HEK293T cells stably expressing FZC18 (batches 1; 4; 5) or empty vector (vector) were seeded at low density and cell number was determined on an 8-day time course by cell counting. (B) Cells were serum-starved for 48 hr and stimulated with 10% FBS twice, as shown *(arrows)*. At each time point, cells were pulsed with 1 µCi/ml ^3^H thymidine for 2 hr before lysis. Incorporated radioactivity is expressed as cpm/µg protein.

We produced zeocin-resistant mass cultures of 293T cells stably expressing FZC18 or empty vector. To avoid clonal variability, we expanded colonies showing different densities of FZC18 (+) cells (Figure 1B). As FZC18 locates preferentially at the cell surface [7], cell permeabilization followed by immunocytochemistry allowed identification of all cells expressing the protein of interest, regardless of protein maturation. Thus, batch #1 showed a lower density of FZC18 (+) cells (Figure 1B) and lower FZC18 expression by immunoblot than batch #4 and #5 cells (Figure 1C). When passaged routinely, FZC18 (+) cells grew more slowly, formed smaller cell plates than vector cells and secreted soluble FZC18 (Figure S1, B and C). Simultaneous detection of N- and C-terminal epitopes in this fusion protein indicated preservation of FZC18 integrity in cells (Figure 1C) and in the medium (Figure S1C). An 8-day time course cell proliferation assay showed that FZC18-expressing cells grew more slowly than vector cells (Figure 2A). ^3H^Thymidine incorporation rates into DNA showed that FZC18 reduces cell proliferation and DNA synthesis (Figure 2B). Throughout the 8-day cell proliferation assay, mitochondrial succinate dehydrogenase activity in living cells (MTT assay) confirmed the decrease in cell growth in FZC18-expressing cells (Figure S2). The decrease in proliferation rates was correlated with the expression levels of FZC18 in the stable cell cultures (Figure 1B). No significant difference in spontaneous cell death was observed in these cells compared to vector-expressing cells by flow cytometry search for subG1, hypo-diploid cells (not shown).

### FZC18 reduces cell sensitivity to soluble Wnt3a

**Figure 3:**
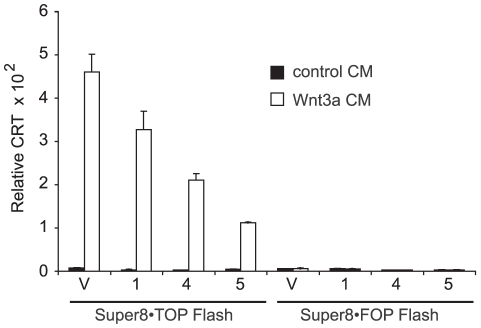
FZC18 reduces cell sensitivity to soluble Wnt3a. HEK293T cell batches stably expressing FZC18 (1; 4; 5) or empty vector (V) were incubated with either 50% control or Wnt3a conditioned medium (CM) for 16 hr before lysis. CRT (β-catenin-T-Cell factor Regulated Transcription) assays using Super8•Topflash or the negative control Super8•Fopflash reporters are representative of three independent experiments performed in triplicate and normalized to Renilla luciferase activity (mean±SD).

Incubation of vector and FZC18-expressing 293T cells with conditioned medium (CM) from L cells secreting soluble Wnt3a (Wnt3a CM) confirmed that FZC18 reduces Wnt3a-induced Wnt signaling (Figure 3), β-catenin stabilization, cyclin D1 promoter activity and protein expression (Figure S3). FZC18-expressing cells showed lower amounts of steady-state β-catenin protein (Figure S3A) and cyclin D1 promoter activity than control cells (Figure S3B). In particular, cyclin D1 protein expression in response to soluble Wnt3a was considerably stronger in vector cells than in FZC18 cells (Figure S3C), indicating that FZC18 abrogates the response to Wnt3a. The dose-response curve to different dilutions of Wnt3a CM showed that FZC18-expressing cells could efficiently build up a CRT response to Wnt3a, in such a way that the higher the concentration of Wnt3a CM, the higher the fold-change in CRT. However, the absolute CRT levels in FZC18-expressing cells were 5 to 8 folds lower than those in vector cells (Figure S3D).

Despite lower pre-Wnt3a and post-Wnt3a β-catenin stabilization and downstream signaling events in FZC18 cells (Figures 3 and S3), a given strength of Wnt3a stimulus induced the same fold-change in CRT both in vector and FZC18 cells (Figure S4). Likewise, it has been recently shown that in a normal cell context, different cell systems respond to Wnt stimulation with similar fold-change despite their different starting and output levels in Wnt/β-catenin signaling [15]. Thus, FZC18 may not impair downstream processing of Wnt stimuli, but it seems to decrease cell sensitivity to Wnt3a, probably by blocking Wnt access to frizzled receptors.

### Soluble FZC18 binds Wnt3a

**Figure 4:**
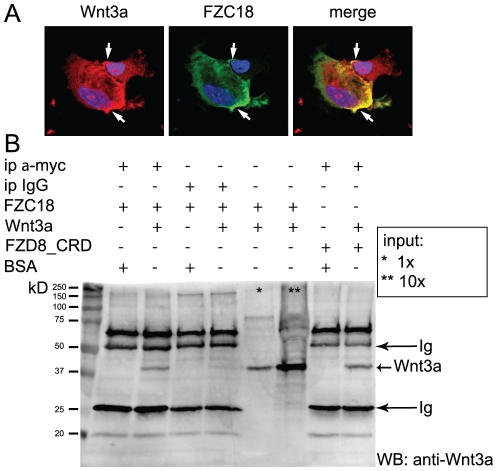
Soluble FZC18 binds Wnt3a. (A) FZC18 colocalizes with Wnt3a at the cell surface *(arrows)*. AT3F1S315 hepatoma cells were co-transfected with FZC18 and Wnt3a vectors. FZC18 *(green)* was detected by anti-myc+FITC-labeled IgG. Wnt3a *(red)* was detected by anti-Wnt3a+biotinylated IgG+streptavidine-Texas red. Cells were not permeabilized. Nuclei were labelled blue with DAPI. Images were acquired using a Leica TCS NT system on a Leica DMB confocal microscope at original magnification ×630. (B) Soluble FZC18 binds Wnt3a. Conditioned medium from HEK293-EBNA cells transiently expressing FZC18 was incubated with recombinant Wnt3a overnight at +4°C. FZC18 was immunoprecipitated with anti-myc and immunoblotted with anti-Wnt3a. Conditioned medium from HEK293-EBNA cells transiently expressing FZD8_CRD-myc was used as a positive control of coimmunoprecipitation. *Ig*, immunoglobulins. *Asterisks* denote inputs. For 10×, FZC18-myc CM was incubated with Wnt3a and then concentrated 10 folds by TCA precipitation.

Co-expression of FZC18 and Wnt3a in non permeabilized AT3F1S315 mouse hepatoma cells [16] followed by confocal microscopy analysis revealed that both proteins colocalized at the cell surface, highlighting cell contacts (Figure 4A). We further confirmed that HEK293T cell clones stably expressed FZC18 at the cell surface by immunostaining with antibodies detecting the N- and C-termini of the FZC18-myc fusion protein (Figure S5A). Moreover, subcellular fractionation confirmed that FZC18 was exclusively detected in the crude membrane fraction (Figure S5B), indicating that the protein is indeed addressed to the secretory pathway.

Using protein extracts from cells cotransfected with both FZC18 and Wnt3a, we previously showed that these ectopically expressed molecules interact [7]. However, cysteine-rich proteins like Wnts and the Frizzled CRDs may get clogged within the secretory pathway [2], leading to high intracellular concentration and spurious interactions. Here, we wished to study these interactions in a cell-free system, using soluble FZC18 and Wnt3a, at low concentrations. Despite easy detection of FZC18-myc by immunocytochemistry, immunoblot detection showed no signal in non-concentrated CM, in contrast to FZD8_CRD-myc, which was detected at high levels (Figure S5C). Concentration of FZC18 CM by 13 folds was required to observe a detectable signal by immunoblot (Figure S5C). For coimmunoprecipitation, we added purified recombinant Wnt3a to non-concentrated FZC18-myc or FZD8_CRD-myc CM. Wnt3a concentration (2.7 nM) was within the physiological range [13], [17], [18], [19], [20]. Under these conditions, both FZC18-myc and FZD8_CRD-myc pulled down Wnt3a (Figure 4B). Accordingly, reverse co-immunoprecipitation revealed that Wnt3a pulled down both FZC18-myc and FZD8_CRD-myc (Figure S5D). Additionally, both precipitation and immunoblot with anti-myc antibody confirmed the presence of soluble FZC18 and FZD8_CRD at the expected amounts in these CM (Figure S5E).

### Extracellular FZC18 inhibits Wnt3a-induced Wnt/β-catenin signaling

**Figure 5:**
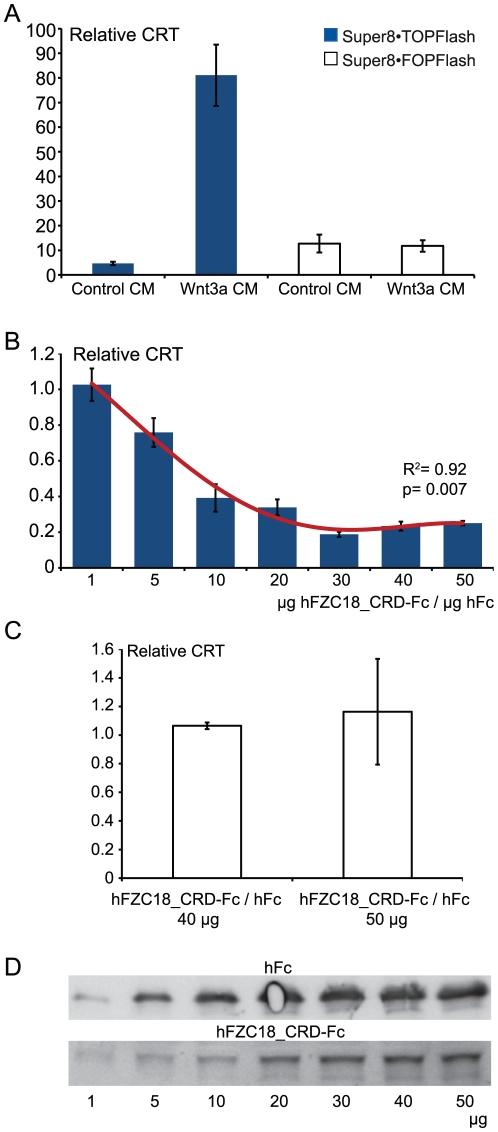
Partially purified FZC18 inhibits Wnt3a-induced Wnt/β-catenin signaling. CRT assay using the β-catenin reporters Super8•Topflash and Super8•Fopflash, as indicated. (A) HEK293-EBNA cells incubated for 16 hr with either 50% control CM or 50% Wnt3a CM. Wnt3a induces an 80-fold increase in CRT. (B and C) Partially purified, Fc tagged human FZC18_CRD (hFZC18_CRD-Fc) dose-dependently inhibits Wnt3a-induced CRT in HEK293-EBNA cells, as shown with Super8•Topflash *(B)* and Super8•Fopflash *(C)* CRT reporters. Cells were incubated for 16 hr with 50% Wnt3a CM that had been pre-incubated overnight on a rotary wheel at +4°C with the indicated concentrations of hFc tag alone (recombinant human Fc from IgG, negative control) or hFZC18_CRD-Fc. Results are shown as mean±SD of hFZC18_CRD-Fc/hFc tag ratios. R^2^ indicates 2^nd^ degree polynomial regression coefficient. Curve fitting is shown by a red line. Super8•Fopflash *(C)* negative control CRT reporters (C) are shown for the highest concentrations of hFZC18_CRD-Fc/hFc. (D) Immunoblots show hFc and hFZC18_CRD-Fc from each sample using anti-Fc tag antibody.

We tested whether the CRD of FZC18 (FZC18_CRD) could effectively inhibit Wnt signaling. FZC18_CRD was cloned in frame with a human Igκ signal sequence and a human Fc tag from IgG. Human FZC18_CRD-Fc preparations with 40% purity were tested for biological activity. As expected, hFZC18_CRD-Fc dose-dependently inhibited Wnt3a-induced CRT (Figure 5). Inclusion of a thrombin cleavage site did not significantly affect Wnt inhibitory activity of hFZC18_CRD-Fc (Figure S6). Taken together, these findings support the concept that FZC18 exerts its biological effects in the extracellular compartment and that the CRD of FZC18 has Wnt inhibitory activity.

FZC18 seems to provide short-range signals, thus working as an SFRP-like molecule [8]. We thus confirmed whether FZC18-expressing cells impact on the microenvironment of adjacent cells and modulate their response to Wnt stimuli. FZC18-expressing cells were co-cultured with parental 293 cells expressing the CRT reporter. Co-cultures were established at different ratios of FZC18 (+) cells to a constant number of reporter cells in the presence of 50% Wnt3a CM (Figure S7). Under these conditions, the response of the reporter cells to soluble Wnt3a was inversely proportional to the number of FZC18 (+) cells in the co-culture system.

### Frizzled 1 and 8 receptors partially rescue the inhibition of Wnt3a-induced β-catenin signaling by FZC18

**Figure 6:**
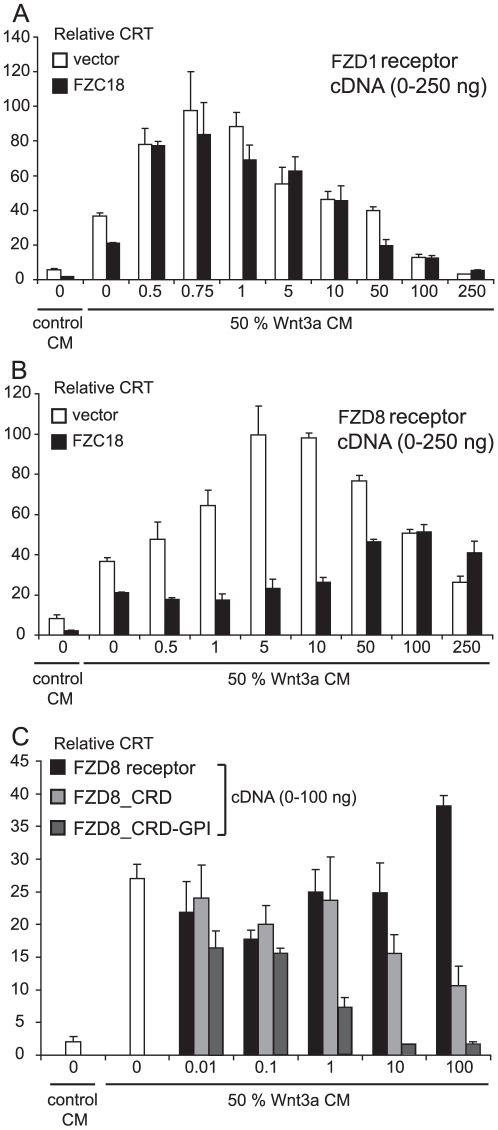
Frizzled 1 and 8 receptors partially rescue the inhibition of Wnt3a-induced CRT by FZC18. CRT reporter gene assays using the β-catenin-TCF responsive reporter Super8•Topflash in HEK293T cells stably expressing FZC18 (A, B and C) and vector (A, B). Twenty-four hours after transfection with the CRT reporter and increasing amounts of either FZD1 receptor (A), FZD8 receptor (B), FZD8 receptor, FZD8_CRD or FZC8_CRD-GPI (C) cDNAs, cells were incubated either with 50% control CM (L) or with 50% Wnt3a CM for 16 hr. Results are representative of three independent experiments performed in triplicate and normalized to Renilla luciferase activity. Error bars represent standard deviations.

We tested whether increasing the availability of cell surface frizzled receptors in FZC18-expressing cells could compete with FZC18, thereby enhancing Wnt signaling. To this end, FZC18-expressing and vector cells were transfected with increasing amounts of full-length FZD1 or FZD8 receptor cDNAs and incubated in the presence of Wnt3a CM (Figure 6, A and B). FZD1 and FZD8 receptor expression led to up-regulation of CRT in both vector and FZC18-expressing cells. Vector and FZC18 cells expressing FZD1 exhibited similar CRT (Figure 6A). By contrast, 100 ng of FZD8 cDNA was required to reach 50% of vector cells' maximal CRT in FZC18-expressing cells, whereas vector cells' maximal activity was obtained with 5 ng of FZD8 cDNA. Interestingly, although lower amounts of FZD1 (0.5 and 0.75 ng) or FZD8 (0.5 to 10 ng) cDNAs activated CRT in a dose-dependent manner, higher amounts of FZD1 (1 to 250 ng) or FZD8 (50 to 250 ng) cDNAs gradually reduced CRT (Figure 6, A and B). Although high doses of FZ receptor cDNA appeared non-specifically inhibitory (Figures 6 and S8), mouse FZD1 and Drosophila FZ3 may behave as antagonists of canonical Wnt/β-catenin signaling [21], [22]. Excessive ectopic stimulation of β-catenin signaling via frizzled receptors may result in saturation of the signal transduction capacity of pathway components downstream to the frizzled receptors. In keeping with this hypothesis, mouse wild-type FZD1 inhibits Wnt signaling less efficiently than does C-terminally deleted mouse FZD1 [21]. These data led us to test a hypothetical synergy between FZC18 and FZ CRDs, capable of binding Wnts but unable to transduce downstream signal.

Using FZC18-expressing cells, we compared the effects of the full-length FZD8 receptor, FZD8_CRD and a FZD8_CRD carrying a C-terminal glycosylphosphatidylinositol (GPI) anchor attaching the CRD at the cell surface (FZD8_CRD-GPI). Within the 10 pg-100 ng range of transfected cDNA, FZD8_CRD-GPI and FZD8_CRD further reduced Wnt signaling in FZC18-expressing cells (Figure 6C). Under the same conditions, full-length FZD8 antagonized the effects of FZC18, increasing CRT (Figure 6C), supporting the data shown in Figure 6B. Effects of full-length FZD8 receptor, FZD8_CRD and FZD8_CRD-GPI on cells expressing the empty vector are provided on Figure S9.

These results show an additive effect of FZC18 and either FZD8_CRD or FZD8_CRD-GPI, the dose-response curve outlining a higher efficacy of FZD8_CRD-GPI. Thus, Wnt/β-catenin signaling can be concomitantly downregulated by different Wnt-binding proteins. As FZD8_CRD is soluble, its cell surface bioavailability may thus be lower than that of FZD8_CRD-GPI, probably resulting in lower Wnt inhibitory activity.

Taken together, these data underline the specificity of the Wnt inhibitory activity of FZC18. As FZD receptors rescued Wnt/β-catenin signaling, but FZD CRDs further enhanced the inhibitory effects of FZC18, these findings indicate that the effects of FZC18 may not result from endoplasmic reticulum toxicity through clogging of the secretion pathway with cysteine-rich proteins.

### FZC18 forms homodimers and binds the CRDs of FZD1 and FZD8 receptors

**Figure 7:**
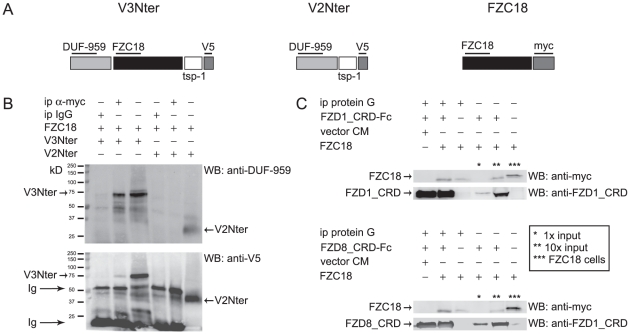
The FZC18 domain homodimerizes and binds FZD1 and FZD8 CRDs. (A) Schematic structure of V3Nter, V2Nter and FZC18 cDNAs. V3Nter and V2Nter correspond to the N-terminal noncollagenous domains of variants 3 and 2 of collagen XVIII, respectively. They share the *DUF-959* domain, a portion of the *tsp-1* (thrombospondin-1) domain and the *V5* tag. Only V3Nter contains the FZC18 domain. The FZC18 vector has a *myc* tag. Thick horizontal lines indicate the antibodies used. (B) FZC18 can homodimerize. FZC18-myc was cotransfected with V3Nter-V5 or V2Nter-V5 in HEK293-EBNA cells. Cell lysates were immunoprecipitated with anti-myc and immunoblotted with anti-DUF-959 *(top)*. The membrane was stripped and re-probed with anti-V5 *(bottom)*. *Ig*, immunoglobulins. (C) Soluble FZC18 binds FZD1_CRD and FZD8_CRD. CM from HEK293-EBNA cells secreting FZC18-myc was incubated with recombinant 100 ng/ml FZD1_CRD-Fc *(upper panel)* or with CM from HEK293-EBNA cells secreting FZD8_CRD-Fc *(lower panel)*. FZD1_CRD-Fc and FZD8_CRD-Fc were immunoprecipitated with protein G magnetic beads, electrophoresed and immunoblotted with anti-myc, anti-FZD1_CRD or anti-FZD8_CRD, as shown. *Asterisks* denote inputs or FZC18 cell lysate, as indicated.

One of the well-known features of frizzled CRDs is their capacity to form homo and heterodimers [4], conferring to SFRPs the ability to bind to frizzled receptor CRDs [5]. To investigate whether these features applied to FZC18, we cotransfected HEK293T cells with both V3Nter-V5 and FZC18-myc or with V2Nter-V5 and FZC18-myc (Figure 7, A and B). V3Nter is a precursor of FZC18 originated by endogenous proteolysis in human tissues [7]. V2Nter-V5 contains the same aminoterminal sequences of C18 as V3Nter, but lacks the FZC18 domain (Figure 7A). As both V2Nter and V3Nter share the DUF-959 domain, V2Nter was used as a negative control. Immunoprecipitation with anti-myc, followed by immunoblotting with anti-DUF-959 or with anti-V5 tag antibodies showed that FZC18 bound V3Nter but not V2Nter. This also excludes the possibility that FZC18 could bind other portions of the V3Nter molecule, such as the DUF-959 or the Tsp-1 domains (Figure 7A).

**Figure 8:**
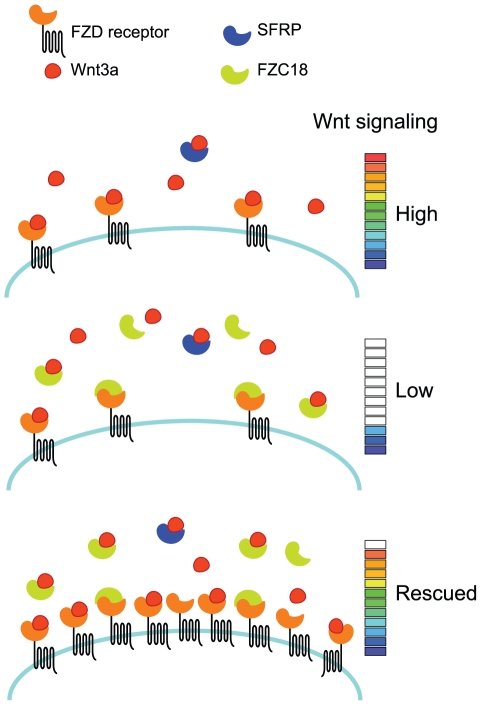
Hypothetical model for the mode of action of FZC18. *High:* in the absence of FZC18, Wnt3a increases β-catenin signaling. *Low:* FZC18 binds Wnt3a and FZD receptors, blocking Wnt/β-catenin pathway activation in an SFRP-like mode of action. *Rescued:* FZC18 inhibitory effects can be partially rescued by increasing the number of FZD receptors at the cell surface.

Next, we tested whether FZC18 could bind the CRDs of FZ receptors. To this end, we used soluble FZD1 or FZD8 CRDs fused to the Fc portion of human IgG (Figure 7C). Adding recombinant FZD1_CRD-Fc or FZD8_CRD-Fc CM to non-concentrated FZC18 CM and immunoprecipitating with protein G coated beads, followed by immunobloting with anti-myc antibody showed that FZC18 could bind FZD1 and FZD8 CRDs. Taken together, the results suggest a model whereby FZC18 could bind both Wnts and FZD CRDs, hampering access of Wnts to the FZD receptors, thus blocking Wnt/β-catenin pathway activation in an SFRP-like mode (Figure 8).

## Discussion

The microenvironment impacts Wnt activity and regulates cell behavior through extracellular molecules that fine tune the response to Wnt stimuli [5], [23]. Proteolysis in human tissues releases active FZC18 and tumors containing high levels of FZC18 show low β-catenin activation levels [7]. FZC18 behaves as an SFRP-like molecule inhibiting in vivo cell proliferation and tumor growth [8]. Although Wnt3a and FZC18 were shown to interact in a cell overexpression system [7], whether the interaction was still active in a cell-free system at physiological concentrations remained unknown. Here, we show that soluble FZC18 binds Wnt3a and the receptors FZD1 and FZD8, and reduces cell sensitivity to Wnt3a. FZC18 inhibitory effects are partially rescued by FZD1 and FZD8 receptors, but enhanced by FZD8_CRD-GPI, a cell-surface-tethered FZD8_CRD chimera. Altogether, the results suggest that FZC18 shifts the sensitivity of cells to Wnt stimuli to a lower pitch, slowing their growth rate.

Although the concentration of soluble FZC18 in the conditioned medium was several fold lower than that of FZD8_CRD, a well-known partner of Wnt3a [6], Wnt3a was efficiently pulled down by FZC18. Accordingly, Wnts bind to FZD CRDs with affinities lower than 90 nM [13], [17], [18], [19], [20]. In the present report, the use of soluble FZC18 at very low concentrations and a Wnt3a concentration within the physiological range (2.7 nM) indicates that spurious interactions of highly concentrated cysteine-rich proteins are unlikely. In addition, we show that FZC18 binds FZD1 and FZD8 CRDs, implying that FZC18 could form nonfunctional complexes with the frizzled receptors, thus acting as a dominant negative inhibitor of Wnt signaling.

The major technical hardship of this work was the low yield of soluble FZC18. Expression of FZC18 using different vectors and mammalian host cell systems, allowing either genomic DNA integration or high copy number episomal replication of target sequences yielded low amounts of soluble FZC18 (not shown). Similarly, generation of isogenic stable mammalian cell lines using Flp recombinase and optimal site-specific genomic recombination failed to increase the yield of soluble FZC18 (not shown). Since we demonstrated that the CRD of FZC18 can form homodimers, we hypothesized that homodimerization could increase yield of soluble protein and expressed human FZC18_CRD sequences in frame with the Fc fragment of IgG in CHO cells. This vector/host combination dramatically increased yield of soluble FZC18_CRD in the medium. Furthermore, partially purified recombinant hFZC18_CRD-Fc inhibited Wnt/β-catenin signaling induced by soluble Wnt3a, confirming that the Wnt inhibitory activity resides within the frizzled CRD of FZC18.

Overexpression of either FZD1 or FZD8 receptors partially rescued Wnt signaling in FZC18-expressing cells. On the other hand, FZD8_CRD-GPI, which remains tethered to the cell surface, enhanced the inhibitory effect of FZC18 more efficiently than FZD8_CRD did, which freely diffuses into the medium. Therefore, competing CRDs may diminish sensitivity to Wnt ligands at the cell surface. Accordingly, recent evidence indicates that FZD receptors may be limiting partners in Wnt signal reception because decreased Wnt/Wg signaling resulting from ubiquitylation of FZD receptors can be rescued by FZD receptor overexpression [24].

Finally, Wnt signaling outputs rely not only on ligand and receptor availability at the cell surface, but also on the microenvironment that can either enhance (such as heparan sulfate proteoglycans) [2] or blur (such as SFRPs, DKKs or FZC18) ligand/receptor interactions. In normal adult human tissues, FZC18 is released by stepwise proteolytic cleavage from a cell surface variant of collagen XVIII [9] [7] which is expressed at low levels. Its expression is up-regulated in liver fibrosis and in small, well-differentiated tumors, but decreases in advanced human liver cancers. Indeed, low FZC18 immunostaining in liver cancers correlates with markers of high Wnt/β-catenin activity [7]. Since the release of biologically active FZC18 is controlled by so far unidentified proteases, it is possible that cell growth or tumor invasion facilitate the local release of FZC18, conveying negative feedback cues to control cell fate. Therefore, further work is necessary to determine if FZC18 works as a cell surface sensor of proteolysis.

Ectopically expressed FZC18 blocks Wnt signaling in an SFRP-like fashion, inhibiting *in vivo* cell proliferation and tumor growth through paracrine signals [8]. However, further work will be necessary to know whether soluble FZC18 inhibits cell proliferation and tumor growth *in vivo*. The present work suggests that soluble FZC18 decreases cell sensitivity to Wnt signals by binding Wnt3a and the receptors Frizzled 1 and 8. Thus, the full repertoire of Wnt ligands and frizzled receptors that interact with FZC18 should be defined, as well as its possible interaction with other molecules of the extracellular matrix. In particular, FZC18 could signal through the non-canonical Wnt pathway to decrease cell proliferation. Comparison of binding affinities of FZC18 with soluble and, as yet unknown, extracellular-matrix-tethered ligands will help better understand the physiological role of this collagen-embedded frizzled CRD.

## Supporting Information

Figure S1 Secretion of FZC18 in the cell medium. (A) Schematic structure showing the FZC18 expression vector. Thick horizontal lines indicate the antibodies used. SP, signal peptide; CRD, cysteine-rich domain; myc, myc epitope tag. (B) Phase contrast images of HEK293T cell batches stably expressing FZC18 (1; 4 and 5) or empty vector (V). Original magnification ×100. (C) Cells were cultured in suspension with neither FBS nor phenol red during 3 days. Conditioned media were collected, dialyzed against deionized water, lyophilized and 150 µg was analyzed by immunoblot using anti-myc and anti-FZC18 antibodies, as indicated. As a positive control, 10 µg of lysate of cells stably expressing FZC18 was used (cells). Arrows indicate FZC18. The asterisk indicates a nonspecific band.
(EPS)

Figure S2 FZC18 inhibits cell proliferation. Proliferation of HEK293T cells expressing FZC18 was assessed by the MTT colorimetric assay measuring mitochondrial activity in living cells on an 8-day time course and is shown as mean±SD of three replications. Results are representative of three independent experiments performed in triplicate.
(EPS)

Figure S3 FZC18 reduces basal level and Wnt3a-induced β-catenin stabilization and cyclin D1 expression. (A) β-catenin assay stabilization assay using anti-β-catenin, anti-non-phosphorylated β-catenin and anti-GAPDH (loading standard) antibodies and (B) cyclin D1 luciferase promoter reporter assay. Cells were incubated with either 50% control or Wnt3a conditioned medium (CM) for 16 hr before lysis. Reporter assays are representative of three independent experiments performed in triplicate and normalized to Renilla luciferase activity (mean±SD). (C) Cells stably expressing FZC18 (batch #5) or vector were incubated with 50% control (−) or Wnt3a (+) CM for 16 hr. Total protein extracts from these cells were analyzed by immunoblot detecting cyclin D1. GAPDH is a loading standard. (D) FZC18 reduces cell sensitivity to soluble Wnt3a. Relative CRT in vector or FZC18 cells (batch #5) incubated with increasing concentrations of control or Wnt3a CM for 16 hr (compare relative CRT values in vector versus FZC18 cells). (E) Aliquots of control (0%) or increasing concentrations of Wnt3a CM (3–100%) from B were immunoblotted with anti-Wnt3a.
(EPS)

Figure S4 Wnt3a induces similar fold-change in Wnt signaling in vector- and FZC18-expressing cells. CRT reporter gene assays using the β-catenin-TCF reporter Super8•Topflash (A) and the negative control reporter Super8•Fopflash (B) in HEK293T cells stably expressing vector or FZC18, as indicated. Twenty-four hours after transfection with the CRT reporters, cells were incubated with serial dilutions of either control CM (from parental L cells) or Wnt3a CM (from L cells secreting Wnt3a) for 16 hr. Results are representative of three independent experiments performed in triplicate and normalized to Renilla luciferase activity. For each dilution of control and Wnt3a CM, fold-changes in CRT were calculated as: (Firefly/Renilla luciferase Wnt3a CM)/(Firefly/Renilla luciferase control CM).
(EPS)

Figure S5 FZC18 is a cell membrane-associated protein which binds Wnt3a in its soluble form. (A) Localization of FZC18 in cell membranes. Immunofluorescent detection of FZC18 N-terminal *(red)* and C-terminal *(green)* epitopes in non permeabilized HEK293T cell batches stably expressing FZC18 (FZC18 #1; #4 and #5) or empty vector (vector). Both epitopes colocalize, outlining cell membranes *(arrows)*. Images were acquired with an Axio Imager M1 and Colibri LED system and AxioVision software (Zeiss) at original magnification ×400 (Vector and cell batches FZC18 #1 and #4) and ×1000 (batch #5). (B) FZC18 is exclusively detected in the crude cell membrane fraction. Cytosol and crude cell membranes from HEK293T cell batches expressing FZC18 (1; 4; 5) or vector (v) were immunobloted with anti-myc. α-tubulin and caveolin-2 are loading standards of cytosol and crude membrane fractions, respectively. (C) Lower yields of soluble FZC18-myc than of FZ8_CRD-myc in transiently transfected HEK293-EBNA cell CM. Both proteins were detected by immunoblot with mouse anti-myc antibody followed by goat anti-mouse peroxidase conjugate. Signal was revealed by enhanced chemiluminescence. Arrows indicate FZC18-myc (∼31 kD) and FZ8_CRD-myc (∼45 kD). Brackets show serum immunoglobulins. The FZC18 blot shows: 1x, whole CM from cells expressing (+) or not (−) FZC18; 13x, trichloroacetic acid (TCA) concentrated whole CM from cells expressing (+) or not (−) FZC18; 60x, Amicon centrifugal concentration of whole CM from cells expressing (+) or not (−) FZC18. The FZ8_CRD blot shows: 13x (−), TCA concentrated whole CM from untransfected HEK293-EBNA cells; 1x (+)(+) whole CM from HEK293-EBNA cells transiently expressing FZ8_CRD from batches #1 and #2; 13x (+)(+) TCA-concentrated CM from HEK293-EBNA cells transiently expressing FZ8_CRD from batches #1 and #2; 60x (+)(+) Amicon centrifugal concentration of whole CM from HEK293-EBNA cells transiently expressing FZ8_CRD from batches #1 and #2. (D) Soluble Wnt3a pulls down FZC18 from the HEK293-EBNA CM. Conditioned medium containing soluble FZC18-myc was incubated with recombinant Wnt3a overnight at +4°C. Wnt3a was immunoprecipitated with monoclonal rabbit anti-Wnt3a and immunoblotted with anti-myc. CM from HEK293-EBNA cells transiently expressing FZ8_CRD-myc was used as a positive control of coimmunoprecipitation. *Ig*, immunoglobulins. (E) CM from HEK293-EBNA cells transiently expressing FZC18 was incubated with recombinant Wnt3a overnight at +4°C. FZC18 was immunoprecipitated with anti-myc. In this figure, the blot from Figure 4D was stripped and immunoblotted with anti-myc. CM from HEK293-EBNA cells transiently expressing FZ8_CRD-myc was used as a positive control of coimmunoprecipitation. *Ig*, immunoglobulins.
(TIF)

Figure S6 Partially purified (40% purity) hFZC18_CRD-Fc proteins containing (#1) or not (#2) a thrombin cleavage site are equally efficient in inhibiting Wnt3a-induced CRT. (A) Schematic representation of hFZC18_CRD-Fc constructs containing (#1) or not (#2) a thrombin cleavage site. Empty Fc expression vector is also shown. (B) Coomassie blue staining. Two µg of protein was run in a reducing 7% PAGE-SDS gel. Theoretical molecular weights of #1 and #2 are 42407 Da and 41792 Da, respectively (8 additional aminoacids from the thrombin cleavage site in #1). Migration of #1 appears lightly faster than that of #2 because pI of #1 (6.35)>pI of #2 (6.23). (C) Partially purified, hFZC18_CRD-Fc proteins containing (#1) or not (#2) a thrombin cleavage site are equally efficient in inhibiting Wnt3a-induced CRT. Both proteins dose-dependently inhibit Wnt3a-induced CRT in HEK293-EBNA cells. CRT assay using the β-catenin reporter Super8•Topflash. Twenty-four hours after transfection with the CRT reporter, cells were incubated during 16 hr with either 50% control CM or 50% Wnt3a CM that had been pre-incubated overnight on a rotary wheel at +4°C with the indicated concentrations of hFc tag alone (recombinant human Fc from IgG, negative control) or hFZC18_CRD-Fc proteins.
(EPS)

Figure S7 Paracrine inhibition of Wnt3a-induced Wnt/β-catenin signaling by FZC18. (A) Schematic representation of the coculture experiment. (B) Relative CRT in reporter 293T cells transfected with the Super8•Topflash reporter and co-cultured with 293T cells stably expressing FZC18 or empty vector at different ratios, as indicated, in the presence of 25% Wnt3a CM for 16 hr. (C) Total protein extracts from these cells were immunoblotted with anti-myc detecting FZC18. The same blot was probed with anti-GAPDH as a loading standard.
(EPS)

Figure S8 Negative control CRT reporter gene assays using the mutated β-catenin-TCF reporter Super8•Fopflash in HEK293T cells stably expressing FZC18 (A, B and C) or vector (A, B). Twenty-four hours after transfection with the CRT reporter and increasing amounts of either FZD1 receptor (A), FZD8 receptor (B), FZD8 receptor, FZD8_CRD or FZC8_CRD-GPI (C) cDNAs, cells were incubated either with 50% control CM or with 50% Wnt3a CM for 16 hr. Results are representative of three independent experiments performed in triplicate and normalized to Renilla luciferase activity. Error bars represent standard deviations.
(EPS)

Figure S9 Wnt3a-induced CRT in HEK293T cells is enhanced by FZD8 receptor and inhibited by FZD8_CRD or FZD8_CRD-GPI cDNA. CRT reporter gene assays using the β-catenin-TCF responsive reporter Super8•Topflash in HEK293T cells stably expressing empty vector. Twenty-four hours after transfection with the CRT reporter and increasing amounts of FZD8 receptor, FZD8_CRD or FZC8_CRD-GPI cDNAs, cells were incubated with either 50% control CM or 50% Wnt3a CM for 16 hr. Results are representative of three independent experiments performed in triplicate and normalized to Renilla luciferase activity. Error bars represent standard deviations.
(EPS)
