# Correction: Inhibition of Wnt/β-Catenin Signaling by a Soluble Collagen-Derived Frizzled Domain Interacting with Wnt3a and the Receptors Frizzled 1 and 8

**DOI:** 10.1371/annotation/bd37b3ad-c242-4e77-9ef3-8e92d1f105f5

**Published:** 2012-03-21

**Authors:** Ismaïl Hendaoui, Elise Lavergne, Heun-Sik Lee, Seong Hyun Hong, Hak-Zoo Kim, Christelle Parent, Nathalie Heuzé-Vourc'h, Bruno Clément, Orlando Musso

There was an error in the funding statement. The correct funding statement is: Institut National de la Santé et de la Recherche Médicale, Institut National du Cancer, Agence Nationale de la Recherche (Emergence-BIO Program 2008), Université de Rennes 1, Région Bretagne, The Korea Research Foundation Grant funded by the Korean Goverenment (KRF-2007-357-C00081). The funders had no role in study design, data collection and analysis, decision to publish, or preparation of the manuscript. 

