# Supplementary figures and images for: Inhibition of Wnt/β-Catenin Signaling by a Soluble Collagen-Derived Frizzled Domain Interacting with Wnt3a and the Receptors Frizzled 1 and 8
Source: PLoS One. 2012 Jan 27;7(1):e30601. doi: 10.1371/journal.pone.0030601 (PMC3267734; doi:10.1371/journal.pone.0030601)

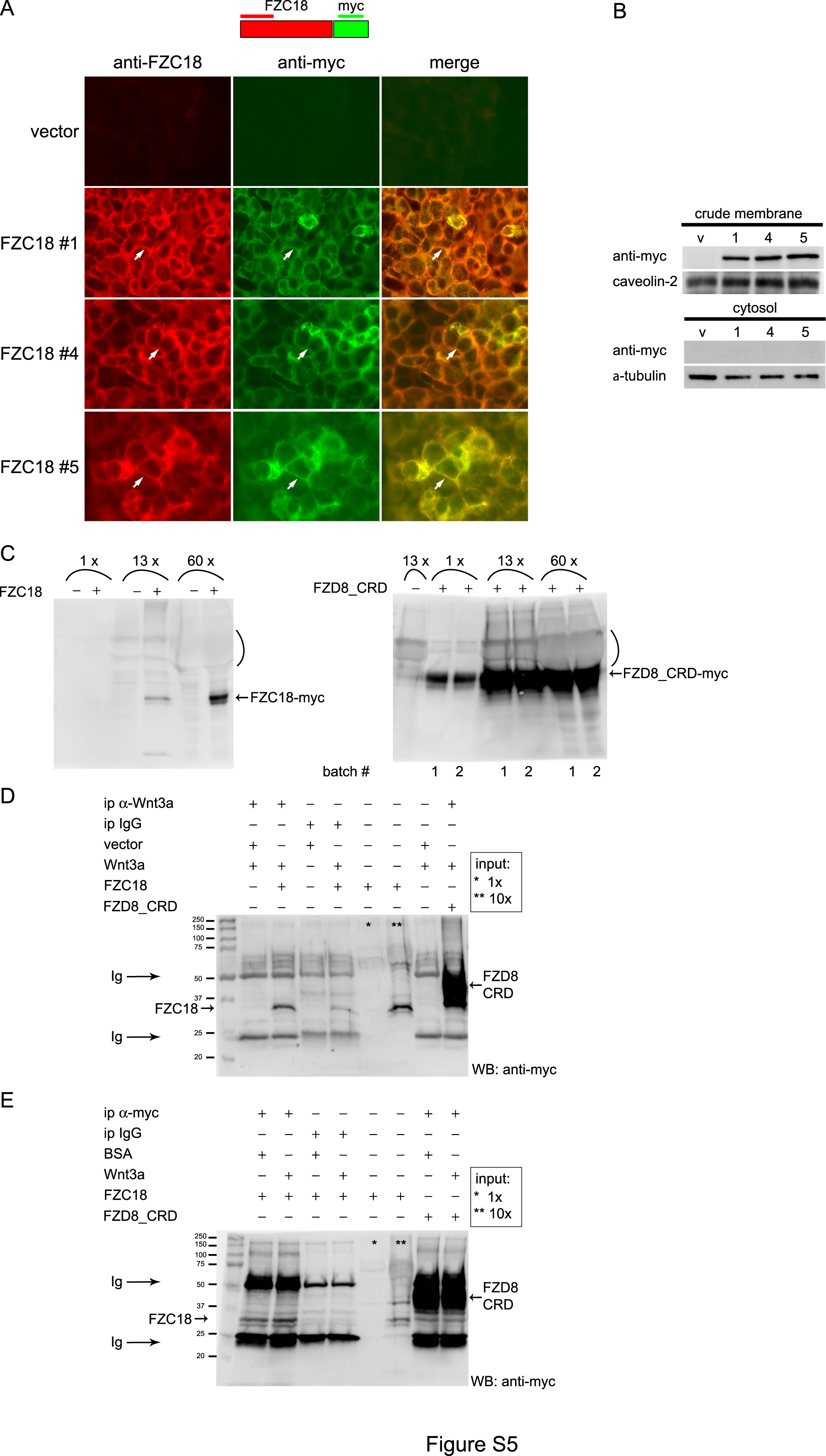

Supplement: Figure S5 — FZC18 is a cell membrane-associated protein which binds Wnt3a in its soluble form. (A) Localization of FZC18 in cell membranes. Immunofluorescent detection of FZC18 N-terminal (red) and C-terminal (green) epitopes in non permeabilized HEK293T cell batches stably expressing FZC18 (FZC18 #1; #4 and #5) or empty vector (vector). Both epitopes colocalize, outlining cell membranes (arrows). Images were acquired with an Axio Imager M1 and Colibri LED system and AxioVision software (Zeiss) at original magnification ×400 (Vector and cell batches FZC18 #1 and #4) and ×1000 (batch #5). (B) FZC18 is exclusively detected in the crude cell membrane fraction. Cytosol and crude cell membranes from HEK293T cell batches expressing FZC18 (1; 4; 5) or vector (v) were immunobloted with anti-myc. α-tubulin and caveolin-2 are loading standards of cytosol and crude membrane fractions, respectively. (C) Lower yields of soluble FZC18-myc than of FZ8_CRD-myc in transiently transfected HEK293-EBNA cell CM. Both proteins were detected by immunoblot with mouse anti-myc antibody followed by goat anti-mouse peroxidase conjugate. Signal was revealed by enhanced chemiluminescence. Arrows indicate FZC18-myc (∼31 kD) and FZ8_CRD-myc (∼45 kD). Brackets show serum immunoglobulins. The FZC18 blot shows: 1x, whole CM from cells expressing (+) or not (−) FZC18; 13x, trichloroacetic acid (TCA) concentrated whole CM from cells expressing (+) or not (−) FZC18; 60x, Amicon centrifugal concentration of whole CM from cells expressing (+) or not (−) FZC18. The FZ8_CRD blot shows: 13x (−), TCA concentrated whole CM from untransfected HEK293-EBNA cells; 1x (+)(+) whole CM from HEK293-EBNA cells transiently expressing FZ8_CRD from batches #1 and #2; 13x (+)(+) TCA-concentrated CM from HEK293-EBNA cells transiently expressing FZ8_CRD from batches #1 and #2; 60x (+)(+) Amicon centrifugal concentration of whole CM from HEK293-EBNA cells transiently expressing FZ8_CRD from batches #1 and #2. (D) Soluble Wnt3a pulls dow [file pone.0030601.s005.tif]
